# Assessing the feasibility of intranasal radiotracer administration for in brain PET imaging

**DOI:** 10.1016/j.nucmedbio.2018.08.005

**Published:** 2018-11

**Authors:** Nisha Singh, Mattia Veronese, Jim O'Doherty, Teresa Sementa, Salvatore Bongarzone, Diana Cash, Camilla Simmons, Marco Arcolin, Paul K. Marsden, Antony Gee, Federico E. Turkheimer

**Affiliations:** a Department of Neuroimaging, Institute of Psychiatry, Psychology & Neuroscience, King's College London, De Crespigny Park, London SE 5 8AF, United Kingdom; b School Biomedical Engineering & Imaging Sciences, 4th floor Lambeth Wing, St Thomas' Hospital, King's College London, London SE1 7EH, United Kingdom; c PET Imaging Centre Facility, King's College London & Guy's and St Thomas NHS Foundation Trust, St Thomas' Hospital, London SE1 7EH, United Kingdom; d Department of Molecular Imaging, Sidra Medicine, Doha, Qatar

**Keywords:** Blood brain barrier, Brain PET imaging, Intranasal, Neuroimaging, Nose-to-brain pathway

## Abstract

**Introduction:**

The development of clinically useful tracers for PET imaging is enormously challenging and expensive. The intranasal (IN) route of administration is purported to be a viable route for delivering drugs to the brain but has, as yet, not been investigated for the delivery of PET tracers. If the intranasal (IN) pathway presents a viable option, it extends the PET imaging field by increasing the number of tracers available for human use.

Here we report the results of a rodent study testing the feasibility of the IN route to administer radiotracers for brain PET imaging.

**Methods:**

We used two different, well characterised, brain penetrant radiotracers, [^18^F]fluorodeoxyglucose ([^18^F]FDG) and [^18^F]fallypride, and aimed to evaluate the pharmacokinetics after administration of the tracers via the intranasal route, and contrast this to intravenous administration. Image acquisition was carried out after tracer administration and arterial blood samples were collected at different time intervals, centrifuged to extract plasma and gamma counted. We hypothesised that [brain region]:[plasma] ratios would be higher via the intranasal route as there are two inputs, one directly from the nose to the brain, and another from the peripheral circulation. To assess the feasibility of using this approach clinically, we used these data to estimate radiation dosimetry in humans.

**Results:**

Contrary to our hypothesis, in case of both radiotracers, we did not see a higher ratio in the expected brain regions, except in the olfactory bulb, that is closest to the nose. It appears that the radiotracers move into the olfactory bulb region, but then do not progress further into other brain regions. Moreover, as the nasal cavity has a small surface area, the extrapolated dosimetry estimations for intranasal human imaging showed an unacceptably high level (15 mSv/MBq) of cumulative skin radiation exposure.

**Conclusions:**

Therefore, although an attractive route for brain permeation, we conclude that the intranasal route would present difficulties due to non-specific signal and radiation dosimetry considerations for brain PET imaging.

## 1 Introduction

The intranasal (IN) route of administration has been employed considerably well for drugs that are needed locally at the site of action, for example, the nasal passage, sinuses or lungs, and in some cases where rapid action is required, i.e., midazolam for epilepsy [1]. Since the 1990s there have been several studies assessing the validity of delivering drugs to the brain via the IN route, by-passing the blood brain barrier (BBB) with varying results [[2], [3], [4], [5], [6]]. However, irrespective of the extensive debates about a ‘direct nose to brain’ pathway [[7], [8], [9], [10]], there has been a surge in the experimental use of IN administration of drugs and biologicals that do not cross the blood brain barrier when administered systemically. Some examples are peptides like insulin, for the treatment of Alzheimer's disease [3], and oxytocin, for various neurodevelopmental disorders [11,12] and schizophrenia [13,14]. Another example, esketamine, an IN preparation of *S*-ketamine for the treatment of unipolar treatment resistant depression [15] that is currently undergoing clinical trials as a viable alternative to intravenous (IV) administration. Although ketamine does cross the blood brain barrier when given IV, this is not a practical route for conditions that require prolonged treatment.

Assuming that IN administration allows brain entry for molecules that do not ordinarily cross the BBB at biologically relevant concentrations, we purported that this route might provide a useful alternative for delivery of tracers for the brain positron emission tomography (PET) imaging. The advantages of IN over IV route for PET imaging includes utilisation of tracers that undergo significant first pass metabolism, tracers that cause changes in blood flow or vasculature and large molecular weight peptides. On the other hand, it is clear that the IN route would also suffer disadvantages including lack of delivery precision, challenging kinetic modelling as well as the limitations related to radiation dosimetry. However, before investigating larger radiolabelled peptides or molecules that do not ordinarily cross the BBB, we decided to take a look at how radiotracers that are known to cross the BBB behave in the instance that we administered them intranasally. We believed that this ‘best-case’ scenario important as it would allow us to assess IN administration in a quantitative manner. Testing a brain impermeable tracer in such a way would present a difficulty in interpretation if there was no discernible signal, for two reasons: first, where the tracer doesn't get into the brain at all, or the second, where it does, but the signal to noise ratio does not allow for a quantifiable image. Therefore, we designed a rodent study and chose two well-characterised, brain-penetrant radiotracers, [^18^F]fluorodeoxyglucose ([^18^F]FDG) and [^18^F]fallypride, that have different tracer characteristics, in order to compare their blood brain barrier permeability and kinetics after IN and IV administrations.

[^18^F]FDG is a radiopharmaceutical glucose analogue (molar mass 181.15 g/mol) that gets taken up as glucose by metabolically active cells. Due to the lack of the 2-hydroxyl functional group however, once up taken it cannot be metabolised through glycolytic pathway, and therefore remains trapped in cells until radioactive decay [16]. When administered IV to healthy volunteers or rodents it accumulates globally throughout the brain. In contrast, [^18^F]fallypride (molar mass 364.45 g/mol) is a high affinity dopaminergic (D_2_/D_3_) receptor antagonist, and as such has the highest uptake in the basal ganglia in normal subjects. Additionally, it also undergoes metabolism and the parent fraction needs to be separated from the radioactive metabolites for accurate quantification.

**Fig. 1:**
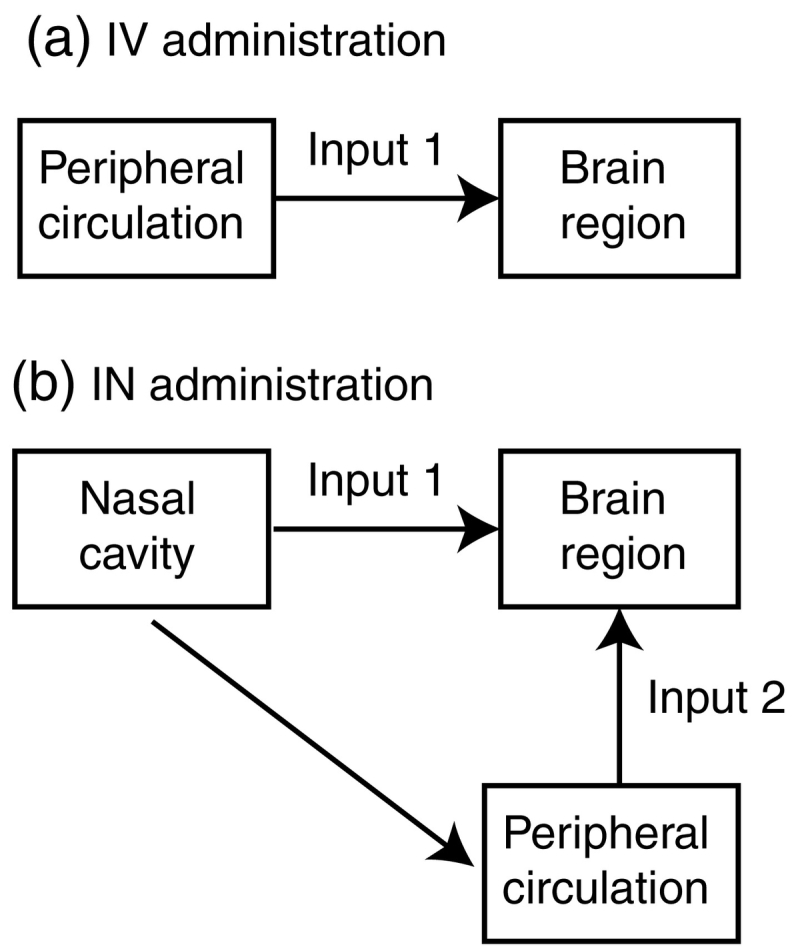
Schematic of the theoretical pathway of radiotracer entry into the brain after (a) intravenous (IV) and (b) intranasal (IN) administration for BBB permeable radiotracers.

How drugs and biologicals move from the nasal cavity into the brain has also been the subject of much scholarship. For details the interested reader should see excellent reviews by Suman [17], Dhuria et al. [9], Lochhead and Thorne [10], Illum [8], and more recently by Crowe et al. [18]. Briefly, molecules from the nose may move through intracellularly through the olfactory and/or trigeminal nerves into the brain, or via paracellular pathways, through spaces between the nerves, to access the brain. As the nasal cavity is highly vascularised, molecules administered to the nose will also be absorbed into blood circulation and the lymphatic system. For those molecules that do not ordinarily cross the BBB, there will be none or negligible access to the brain via peripheral circulation. However, for molecules that can cross the BBB, there would thus be two pathways to the brain: one directly from the nose to the brain via one of the aforementioned routes, and the second via the peripheral circulation [18,19] (Fig. 1).

In this study, as we have selected two BBB permeable tracers, [^18^F]FDG and [^18^F]fallypride, we expected that both tracers will access the brain ‘directly’ via the nasal cavity as well as ‘indirectly’ after entering the peripheral circulation. Therefore, we hypothesise that the radiotracer concentration, relative to plasma, would be higher for IN than for IV administration in the target brain region. More specifically, after IN administration, we would expect a higher plasma-corrected, quantitative uptake of [^18^F]FDG globally in the brain, and in the basal ganglia for [^18^F]fallypride, compared to IV administration, which would indicate that IN administration is a viable for brain PET imaging.

## 2 Materials and methods

### 2.1 Rats

All experiments were conducted in accordance with the Home Office Animals (Scientific procedures) Act, UK, 1986 and were approved by the King's College London ethical review committee. Adult (62.1 ± 6.7 days postnatal), male Sprague–Dawley rats (297 ± 44 g, Charles River, UK) were group-housed at 21 ± 1 °C in a 12-h light:dark cycle and with ad libitum access to standard rat chow and drinking water. Animals were housed for a minimum of one week prior to any experimental procedures. For the [^18^F]FDG experiments, the rats were fasted 18 h prior to the experiment. We used 4–7 per group for the [^18^F]FDG experiments and 5 rats per group for the [^18^F]fallypride experiments. The reporting of this study complies with the Animal Research: Reporting in vivo experiments (ARRIVE) guidelines (https://www.nc3rs.org.uk/arrive-guidelines) [20,21].

### 2.2 Radiochemistry

[^18^F]Fallypride was prepared by reacting the starting material tosylate (2–3 mg) with resolubilized K[^18^F]F/K_222_/K_2_CO_3_ in acetonitrile (1 mL) at 80 °C for 15 min using the Eckert & Ziegler Modular-lab system. The starting material tosyl-fallypride (2.0 mg) was purchased from ABX. [^18^F]Fallypride was purified by a semipreparative HPLC method using Onyc monolithic C18 (100 × 10 mm) and eluent 16:84 ethanol:H_2_O (NaH_2_PO_4_ 0.05 M). HPLC product fraction was filtered through a sterile membrane filter into a vented sterile sample vial containing 5 mL of saline. The average radiochemical yield was 20% at end-of-delivery (EOD). A sample of solution containing [^18^F]fallypride was analysed by an analytical HPLC method using Luna C18 (250 × 4.6 mm, 5 μm) and eluent 52:48 H_2_O (0.1 M Ammonium formate and 0.052 mM acetic acid):acetonitrile for the determination of molar activity, radiochemical purity (>99%) and chemical purity (>99%). The molar activity ranged from 179 ± 76 GBq/μmol, decay corrected to end-of-delivery.

[^18^F]FDG was synthesized using 1,3,4,6-tetra-*O*-acetyl-2-O-trifluoromethanesulfonyl-beta-d-mannopyranose as precursor and synthesized on a GE TracerLab MX Synthesizer as previously described [22].

### 2.3 Surgical procedure

Anaesthesia was induced with isoflurane (5% in oxygen) and maintained on 2% isoflurane in oxygen at a flow rate of 1 mL/min. The femoral vein and artery were surgically exposed and a cannula was placed into the vein (Linton Instruments #BPE-T60) and the artery (Linton Instruments # BPE-T50). The rats were then randomly allocated to receive the tracer either intranasally or intravenously.

### 2.4 Tracer administration

For the IN administration, a 0.3 mL insulin syringe (BD #ND890) was fitted with a PE10 cannula (15 mm) such that it covered approximately 3 mm of the needle tip. 20 μL of radiotracer was administered into each nare as rapidly as possible (approximately within 1 min) and the syringe radioactivity measured before and after administration to calculate the injected dose.

Pilot studies showed that a 30 min after IN administration was the optimal time-point in terms of image resolution for [^18^F]FDG. Therefore, the average time for the start of the PET acquisition was 33 ± 0.8 min after IN administration. Although our pilot study ascertained that [^18^F]fallypride should be IN administered immediately before acquisition, our actual time delay between administration and the start of acquisition was 10.3 ± 2.6 min. This was due to the time taken to move and position the animal in the scanner, having administered it on the bench (to avoid scanner contamination).

**Fig. 2:**
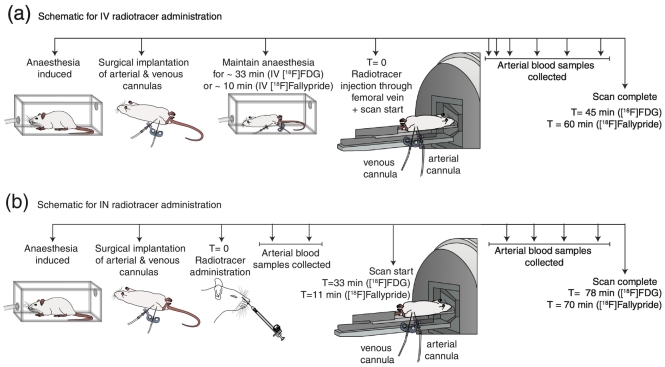
Schematic illustration of the procedure of radiotracer administration. (a) The methodology for the intravenous (IV) tracer administration and (b) the methodology for the intranasal (IN) tracer administration.

The IV administration of radiotracers was carried out via the femoral vein cannula and there was no lag between the injection and start of the acquisition. However, prior to the tracer administration, these ‘IV rats’ were kept under anaesthesia for an additional period of time (~33 min for [^18^F]FDG and ~10 min for [^18^F]fallypride) to match the total time spent under anaesthesia for ‘IN rats’ (Fig. 2).

### 2.5 PET/CT acquisition and reconstruction

Rats were placed in a supine position and scanning was carried out using a BioScan nanoPET-CT^Plus^ (Mediso, Hungary) scanner. Dynamic PET scans were performed on a single volume of interest, and images were acquired over 45 min and 60 min for [^18^F]FDG and [^18^F]fallypride, respectively. The scans were obtained at 400–600 keV energy window, 5 ns coincidence time and coincidence mode of 1–5. CT scan was performed at standard frame resolution (512 × 512 pixels), 55 kVP tube voltage, 600 ms of exposure time and 360° projections.

Reconstruction was carried out using ordered subset expectation maximisation (OSEM) iterative reconstruction algorithm (propriety software, Mediso Ltd.); binning intervals 4 × 15 s; 1 × 60 s; 1 × 180 s; 2 × 300 s; 3 × 900 s for [^18^F]fallypride and 4 × 15 s; 1 × 60 s; 1 × 180 s; 2 × 300 s; 2 × 900 s for [^18^F]FDG. Corrections for decay, randoms, crystal dead time, detector normalisation and attenuation correction were implemented. Images were reconstructed with a voxel size of 0.25 × 0.25 × 0.25 mm^3^ for CT, and 0.4 × 0.4 × 0.4 mm^3^ for PET. The PET and CT images were co-registered automatically.

### 2.6 Image analyses

Images were analysed using VivoQuant 2.0 (Invicro LLC) software. The 3D rat brain atlas template was used to determine brain region (11 regions including whole brain) specific tracer concentrations. For SUV quantification in the throat and nose the image quantification was carried out using PMOD version 3.7 (PMOD Technologies LLC, Switzerland).

Standardised uptake values (SUV) (g/mL) were calculated by dividing the image derived concentration with the ratio of the injected dose to the body weight. For the assessment of dose remaining in the nasal cavity (Fig. 6), the image derived SUV was normalised, whereby the highest value was fixed at 100%.

### 2.7 Blood sampling and processing

Arterial blood samples were collected in EDTA lined blood tubes (BD #367839) at specific time intervals after the administration of the tracer. 10 μL of blood was removed for gamma counting before the sample was centrifuged (10,000 ×*g*, 5 min, 4°) to separate the plasma. Additionally, in case of [^18^F]fallypride, 50–100 μL of plasma was removed and added to an equal volume of acetonitrile to precipitate the proteins. The sample was centrifuged (10,000 ×*g*, 5 min, 4°) to separate the supernatant from the protein precipitant. 100 μL of the supernatant was removed and injected into a high-performance liquid chromatography (HPLC) system (Agilent 1200 series) to separate the metabolites from the parent compound. The gradient used was as described in Peyronneau et al. [23]. Briefly, a Luna® 5 μm C18(2) 100 Å, 250 × 4.6 mm column (Phenomenex# 00G-4252-E0) was used. A linear gradient of 0–70% B over 20 min was applied using the mobile phases 0.1% *v/v* trifluoroacetic acid in water (A) and 0.1% *v/v* trifluoroacetic acid in acetonitrile (B) at a flow rate of 1 mL/min and 1 mL fractions were collected for the duration of the run. The fractions were counted, decay corrected and plotted in GraphPad Prism version 6 h (San Diego) in order to calculate area under the curve.

The plasma radioactive counts obtained were converted to MBq using a calibrated conversion scale, and %injected dose/g was calculated (%ID/g) for each time point.

### 2.8 Calculation of Ratio for comparison between IN and IV administration routes

As the scanning start time was not identical for IN and IV radiotracer administration (see Fig. 2), all the imaging data, for both [^18^F]FDG and [^18^F]fallypride, were realigned and decay-corrected to the time of tracer injection for each animal.

**Fig. 3:**
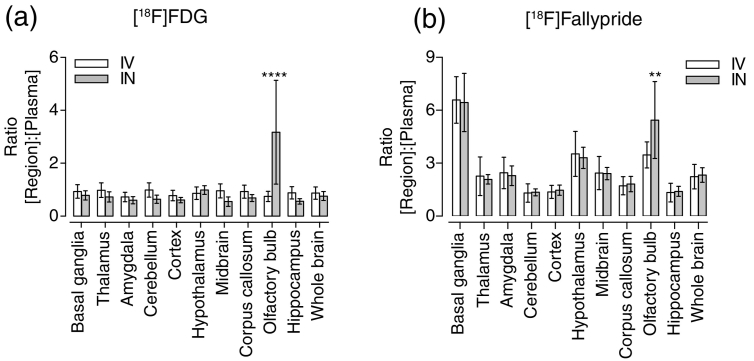
The ratio of the regional radiotracer concentration to the plasma concentration is shown on the y-axis for (a) [^18^F]FDG and (b) [^18^F]fallypride for both intravenous (IV), in white, and intranasal (IN), in grey, administration. The bars represent the mean values and the error bars show the standard deviation. Two-way ANOVA showed no statistically significant difference between the two routes. Only the olfactory bulb shows a significantly higher uptake for both [^18^F]FDG (*p* < 0.0001) and [^18^F]fallypride (*p* = 0.0073) through intranasal compared to intravenous administration.

**Fig. 4:**
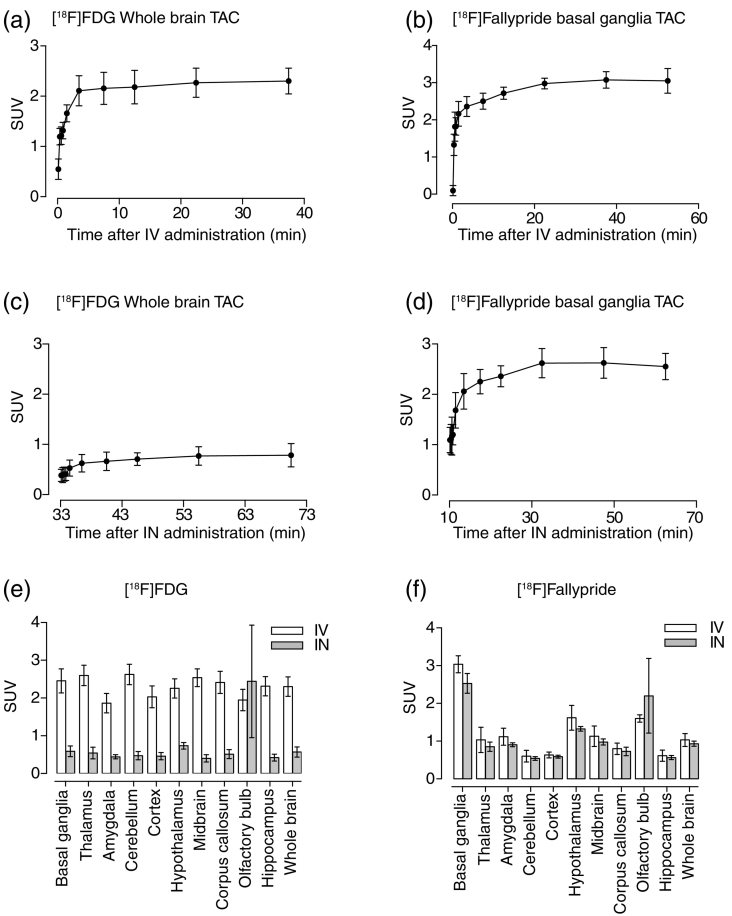
Time activity curves for intravenous (IV) administration of [^18^F]FDG for the whole brain (a), and [^18^F]fallypride for the basal ganglia (b) are shown. The y-axis depicts the standardised uptake value (SUV) and time on the x-axis. The scan start time and radiotracer administration time are identical in case of the IV route. (c) and (d) show the time activity curves for intranasal administration of [^18^F]FDG for the whole brain and [^18^F]fallypride for the basal ganglia, respectively. The y-axis depicts the standardised uptake value (SUV) and time after intranasal administration (min) is plotted on the x-axis. Please note that the scan start time differs from the radiotracer administration time (~33 min and ~11 min prior to scan start for [^18^F]FDG and [^18^F]fallypride, respectively). (e) and (f) show the SUV comparison, corrected for the delay, between intravenous (IV) in white, and intranasal (IN) administration in grey for [^18^F]FDG and [^18^F]fallypride, respectively. The bars represent the mean values and the error bars show the standard deviation.

To normalise the tracer tissue uptake with the blood tracer supply, the ratio between tissue and plasma tracer concentration was hence calculated for each region of interest (ROI) at 30–45 min after tracer injection for [^18^F]FDG, and the time frames between 15 and 60 min after tracer injection for [^18^F]fallypride (Fig. 3, Fig. 4).

### 2.9 Dosimetry calculations for extrapolation to humans

Dosimetry calculations were based on the administration of radioactive ^18^F-labelled compounds into the nasal cavity as extrapolated from the [^18^F]fallypride rat experiments. The most common methods to calculate radionuclide dosimetry involve the use of the Medical Internal Radiation Dose (MIRD) schema. However, as the feasibility study described in this work involves a source of radiation in the nasal cavity (not a part of the standard MIRD schema), this method cannot be applied and thus other methods were investigated.

Previous work using Monte Carlo simulations has determined absorbed skin dose rate conversion factors for a range of radionuclides, which subsequently allow the calculation of cumulated absorbed dose to the basal layer of the skin after a contamination event [24,25]. The cumulative skin dose can be expressed as:

(1) $$D_{T}=D\times \frac{A_{D}\left(1-e^{-\lambda_{\mathrm{eff}}\Delta T}\right)}{\lambda_{\mathrm{eff}}e^{\left(-\lambda_{\mathrm{eff}}\Delta T\right)}}$$

where *D*_*T*_ represents total cumulated skin dose over an area of 1 cm^2^ (mSv), *D* represents the skin dose rate of a 1 cm^2^ contamination, averaged over an area of 1 cm^2^ (mSv/h/kBq); *A*_*D*_ is the mean measured activity over 1 cm^2^ (kBq); *λ*_*eff*_ is the effective decay constant (=ln(2)/*T*_1/2eff_ approximated here as standard physical decay only) and Δ*T* is the total exposure time (*h*). Extending the contamination methodology of the skin to the approximation of skin inside the nasal cavity, and on the assumption that upon IN administration [^18^F]fallypride covers the entire nasal cavity of an area of approximately 160 cm^2^ [26], cumulative absorbed skin dose can thus be calculated via Eq. (1).

A conversion factor from previous work [27] gives an absorbed skin dose rate value for ^18^F-based radiopharmaceuticals of 1.95 mSv/h per kBq/cm^2^. In the case of IN administration of [^18^F]fallypride, the optimal uptake time from the rat experiments was 22.5 min. Dynamic scanning then takes approximately 40–60 min. For these preliminary dosimetry estimations, we thus assumed that the radiotracer was fixed in place in the nasal cavity for 1 h and calculated the skin dose accordingly.

### 2.10 Statistics

All data shown is mean ± standard deviation, unless otherwise mentioned. All statistical analyses were carried out using GraphPad Prism (version 6.0 h), and *p* ≤ 0.05 was considered to be statistically significant. Two-way analysis of variance (ANOVA) with the Sidak correction for multiple comparisons was used to ascertain statistical significance where appropriate.

For [^18^F]fallypride metabolism, data were analysed by using a nonlinear curve fit for one-phase decay and a comparison of fits was carried out to detect differences. Differences in plasma radiotracer concentrations via the two routes were assessed using the Student *t*-test.

## 3 Results

### 3.1 Higher plasma corrected uptake after IN administration in the olfactory bulb

Comparing regional brain uptake for both tracers revealed that, relative to plasma radiotracer concentration, there were no significant differences between IV and IN administration, except in the olfactory bulb.

We had hypothesised that due to two inputs, IN administration should show greater uptake than IV administration, into the target brain areas regions (whole brain and basal ganglia for [^18^F]FDG and [^18^F]fallypride, respectively). However, the data demonstrate that this is not the case. Statistical testing revealed no significant effect of route of administration in either cases, [^18^F]FDG (*p* = 0.62) and [^18^F]fallypride (*p* = 0.38). Additional comparisons between the different brain regions (Fig. 3) for both routes showed that only the olfactory bulb had significantly higher uptake after IN administration.

Taken together, this suggests that in the given time frame, the uptake in the whole brain for [^18^F]FDG, and in the basal ganglia for [^18^F]fallypride, is entirely dependent on the peripheral circulation, except in the olfactory bulb. The nasal input into the whole brain is either negligible or does not occur, in spite of higher uptake into the olfactory bulb.

### 3.2 SUV comparisons

Fig. 4(a–b) shows the time activity curves for IV administration of [^18^F]FDG and [^18^F]fallypride, in the whole brain and basal ganglia respectively, and Fig. 4(c–d) shows the same for IN radiotracer administration. With both [^18^F]FDG and [^18^F]fallypride, we obtained quantifiable images after IN administration. However, the graphs are not directly comparable, as mentioned previously under the methods section. Therefore, bar graphs are plotted (Fig. 4e–f) showing SUV comparisons, corrected for time, between IN and IV administrations. [^18^F]FDG (Fig. 4e) showed a significantly lower uptake (*p* < 0.0001) in case of the IN route, as expected. Only the olfactory bulb showed equivalent uptake, i.e., no significant difference (*p* = 0.25) in case of both routes, IV and IN.

Interestingly, in the case of [^18^F]fallypride, although there is a trend of overall decreased uptake via IN administration, the two routes are not statistically different (*p* = 0.062), i.e., both administration routes are akin with regards to the brain uptake (Fig. 4f). Regionally, the olfactory bulb shows a significantly higher uptake (*p* = 0.0082) for IN administration, whereas the basal ganglia shows a slight decrease in uptake (*p* = 0.042), but the difference with IN administration, overall, is not as apparent as in the case of [^18^F]FDG (Fig. 4e).

However, as the ratio ([brain uptake]:[plasma concentration]) was also independent of route of administration (Fig. 3b), we expect that correspondingly, the plasma concentration of the [^18^F]fallypride should be equivalent for both administration routes.

### 3.3 Plasma radiotracer concentrations

**Fig. 5:**
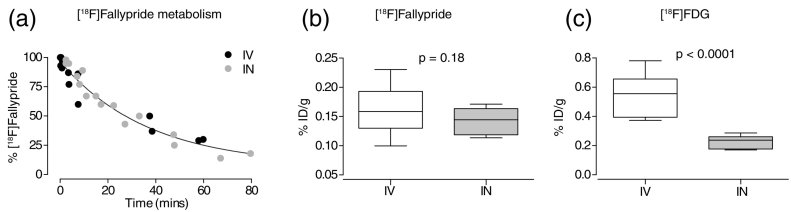
(a) [^18^F]fallypride metabolism over time. The y-axis shows the % of parent fraction in the plasma over time in minutes (x-axis), after intravenous and intranasal administrations. A single exponential decay curve fits both plots as no differences were observed in the metabolism of [^18^F]fallypride when administered intranasal compared to intravenous. (b–c) Graphs showing the average plasma levels as % injected dose per gram (%ID/g) on the y-axis of (b) [^18^F]fallypride at time 48.3 ± 11.3 min (intravenous) and 48.4 ± 10.8 min (intranasal) after tracer administration and (c) [^18^F]FDG at 46.7 ± 9.7 min (intravenous) and 46.7 ± 7.5 min (intranasal).

When we compared plasma radiotracer concentrations at the same time points as those used for SUV and ratio calculations, we observed no significant differences in metabolism (Fig. 5a) and in the plasma concentration of [^18^F]fallypride (Fig. 5b) via the IN and IV routes. Plasma [^18^F]FDG concentration, however, was significantly lower (*p* < 0.0001) when administered IN (Fig. 5c).

Taken together, this suggests that in the case of intranasally administered [^18^F]fallypride, there is substantial, rapid absorption into the peripheral circulation. This is not the case with [^18^F]FDG, for which only a significantly smaller proportion gets absorbed into the periphery. Therefore, after IN administration, the brain uptake (excluding olfactory bulb) in case of [^18^F]fallypride is almost entirely supplied from the peripheral circulation rather than the nasal cavity (see Fig. 1).

### 3.4 Activity remaining in the nasal cavity after IN administration

**Fig. 6:**
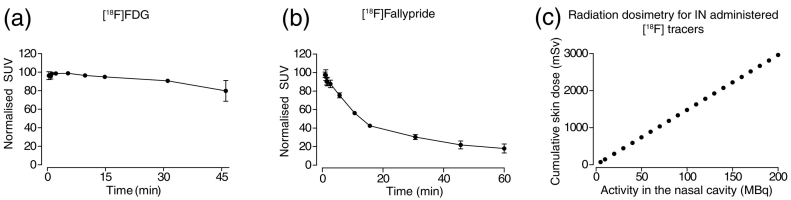
Activity remaining in the nasal cavity over time for (a) [^18^F]FDG and (b) [^18^F]fallypride are graphed. The y-axis shows normalised image derived SUVs, and the x-axis shows the length of scan in min. (c) Total cumulative skin dose to an area of 160 cm^2^ as a result of activity remaining in the nasal cavity, as calculated via Eq. (1).

Fig. 6 shows the image derived, normalised TACs for the radioactivity retained in the nasal cavity for the duration of the scan. In case of [^18^F]FDG, the tracer reduces by only ~30% over the 45 minute scan duration (Fig. 6a), whereas for [^18^F]fallypride the reduction from the start to the end of the 60 minute scan is ~80%. This is in line with the previous results showing that a significant proportion of [^18^F]fallypride gets absorbed into the peripheral circulation, whereas [^18^F]FDG remains trapped in the nasal cavity.

## 4 Dosimetry extrapolations from

As described previously in the methods (see section on ‘Dosimetry calculations’), Eq. (1) can be used to calculate cumulative skin dose. Assuming a worst-case scenario, that the radiotracer remains in place for 1 h, the cumulative skin dose can be calculated depending on the activity that remains trapped in the nasal cavity. Fig. 6c demonstrates that for a known activity remaining in the nasal cavity, the absorbed radiation dose increases linearly. For example, for a trapped activity of 200 MBq, a cumulative skin dose, assuming 160 cm^2^ as the total surface area exposed in humans [26], a radiation dose of 3000 mSv can be reached.

## 5 Discussion

This novel, pilot study examined whether the IN route is a viable option for the administration of radiotracers to image the brain. Although other studies have looked at drug administration to the brain using radiolabelled compounds, this is the first study to look at the feasibility of this administration route for brain PET imaging. The utility of this route of administration would be immense as crossing the blood brain barrier poses a significant barrier to the development of new PET radiotracers.

Based on this study, however, it can be reasoned that the IN radiotracer administration route would not be suitable for brain PET imaging in humans. We hypothesised that the plasma corrected uptake should be higher in the target regions for IN administration to be viable. As we observed a higher uptake only in the olfactory region, but not in the target region, it seems that radiotracer uptake into the brain was non-specific and localised to the area closest to the nasal cavity. In man, by virtue of a larger brain, the distance between the olfactory bulb and others is even larger than in rodents. Even if we did assume some movement through the brain parenchyma, or flow, the time taken to reach specific targets might be too slow for PET imaging.

Secondly, the cumulative skin radiation doses attained in order to obtain suitable image quality for quantification might be prohibitively high and deemed unsafe. Although there are no formal radiation limits for exposure to patients (provided the exposure is ethically justified), the International Committee on Radiation Protection recommends an equivalent skin dose limit of 500 mSv per year in order to minimise deterministic effects (tissue reactions known to increase with radiation dose). From our simulations of radiation dosimetry in this work, this 500 mSv limit would be reached with a trapped activity in the nasal cavity of approximately 35 MBq. Based on extrapolation of the rat data, where only 0.2% of the administered [^18^F]fallypride reaches the basal ganglia, 35 MBq administered intranasally may not give a quantifiable signal in the basal ganglia. Therefore, the amount of radioactivity that can be safely administered intranasally will likely compromise the image quality, making brain PET imaging unlikely. It is likely that we have been overcautious in making the dosimetry estimates as a proportion of radiotracer does move out of the nasal cavity into the circulation. We estimated the worst-case scenario where a ^18^F-labelled tracer remains in the nasal cavity for 1 h. However, our data showed that this was not necessary the case (Fig. 6a–b) and that although both [^18^F]FDG and [^18^F]fallypride remain in the nasal cavity, the latter would have a lower cumulative skin dose compared to [^18^F]FDG, as it gets rapidly absorbed into circulation. Efforts should be made to investigate this further using Monte Carlo simulations of radiation transport and dosimetry and computerised models of PET detection.

A handful of studies have administered radiopharmaceuticals intranasally into human subjects with different aims. A few have assessed the distribution of ^11^C-labelled drugs when given via an intranasal spray and used activities in the range of 2.5–12 MBq [28,29]. One study by Okuyama, administered 148 MBq of [^99m^Tc] to two volunteers intranasally to determine brain uptake as a surrogate marker for the integrity of the BBB [30]. He observed that there was uptake in one volunteer but not in the other, and therefore concluded that the integrity of the BBB was comprised in the volunteer that showed nose to brain uptake.

**Supplementary Fig. 1:**
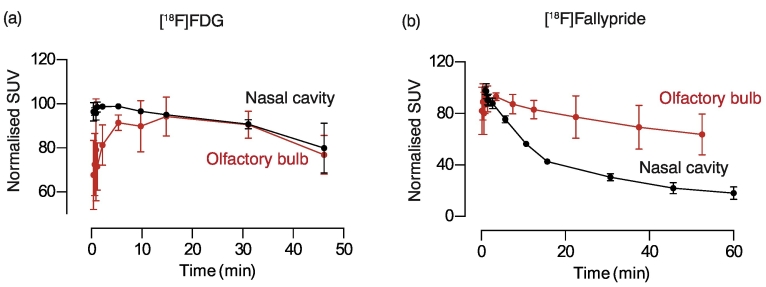
Graphs showing the time activity curves for (a) [^18^F]FDG and (b) [^18^F]fallypride for the nasal cavity (black) and olfactory bulb (red). The SUV has been normalised where the highest activity is considered to be 100%.

**Supplementary Fig. 2:**
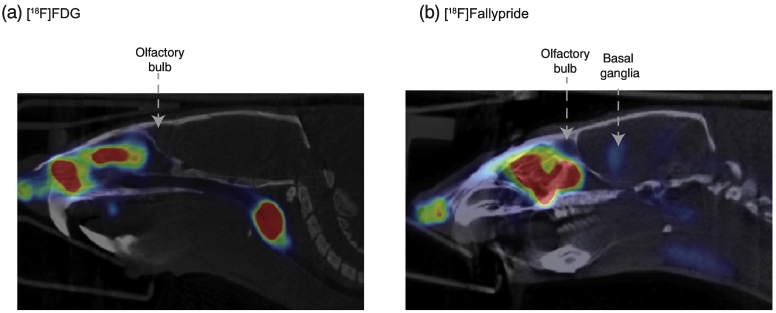
Figure shows representative images of the lateral view of the brain after intranasal administration of (a) [^18^F]FDG and (b) [^18^F]fallypride. The olfactory region is indicated with the arrow in both cases, as well as the basal ganglia in case of [^18^F]fallypride.

One possible technical concern with our pilot study could be that the effects we were observing in the olfactory bulb were due to spill-over effect from the nasal cavity, rather than the uptake in the olfactory bulb, as the distance between the two is very small. Nevertheless, it is unlikely that the signal we observe in the olfactory bulb due to spill over in its entirety. Indeed, the kinetics of the radiotracer uptake and clearance in the olfactory bulb were different from that of the nasal cavity (Supplementary Fig. 1). Had the effect been solely due to spill-over, the kinetics for both should have been identical, which was not the case. Moreover, there is evidence of uptake only in the olfactory bulb, and no other brain regions, in another study using a radiolabelled peptide [2]. The study showed that the signal in the olfactory bulb could be modulated by administering the peptide with or without cyclodextrin (an adjuvant used to increase brain uptake), without changing the total amount present in the nasal cavity [2]. Representative images showing the lateral view after IN administration of [^18^F]FDG and [^18^F]fallypride are shown in Supplementary Fig. 2.

An interesting observation was that the regional brain uptake (excluding the olfactory bulb) for [^18^F]fallypride was relatively similar for both the IN and IV routes (Fig. 4f), but not in the case of [^18^F]FDG (Fig. 4e) where IN administration resulted in a significantly lower uptake. This divergence may be attributed to the difference in cellular dynamics of the two tracers, i.e., [^18^F]FDG gets trapped inside cells whereas [^18^F]fallypride does not. Therefore, a plausible explanation is that a large proportion of [^18^F]FDG gets metabolically trapped in the cells of the nasal cavity and therefore has significantly lower blood levels in case of IN administration (Fig. 5c). However, [^18^F]fallypride is not restricted by cellular trapping, and therefore seems to be rapidly absorbed into peripheral circulation (Fig. 5b) and then from the periphery into the brain tissues, resulting in comparable SUVs between the IN and IV routes. This suggests that cell residence time may also play an important role in the delivery of molecules from the nasal cavity into the brain. How this might affect the IN delivery of other molecules to the brain, especially ones that do not ordinarily cross the blood brain barrier, remains to be tested.

In this study we used tracers that are already known to cross the blood brain barrier. As this is the first study of its kind, we believed it a good starting point to work out kinetics and image quality before moving to tracers that do not normally cross the blood brain barrier. This work shows that even for tracers that are BBB permeable, and have similar physicochemical properties, IN administration can show widely varying results. Indeed a study looking at all quantitative intranasal drug delivery experiments carried out between 1970 and 2014 (a total of 73 publications) showed widely variable results [31]. Moreover, their analyses showed that there was no correlation between the physiochemical properties of the molecules and uptake into the brain [31]. To add to the complexities of IN administration, some studies have found evidence of higher uptake in non-olfactory regions, such as the midbrain and cerebellum [32], whereas other, like us, have found a higher uptake only to the regions closest to the nasal cavity [2,33]. It appears that there is no consensus to predict which molecules are likely to enter the brain, and how far beyond the olfactory bulb they might reach. Consequently, each molecule requires individual quantitative verification if it is to be administered via IN administration.

To summarise, in spite of being a valuable route of administration for drugs that do not cross the BBB, this study concludes that the IN administration route is not a generally suitable route for administering PET tracers in humans, although one cannot exclude that this route may work for specific molecules.

The following are the supplementary data related to this article.
